# Current management of *Staphylococcus aureus* bacteremia in a Japanese university hospital

**DOI:** 10.20407/fmj.2024-010

**Published:** 2024-08-28

**Authors:** Shogo Hanai, Masashi Yokose, Yukinori Harada, Yohei Doi, Taro Shimizu

**Affiliations:** 1 Departments of Microbiology and Infectious Diseases, Fujita Health University, School of Medicine, Toyoake, Aichi, Japan; 2 Department of Diagnostic and Generalist Medicine, Dokkyo Medical University, School of Medicine, Shimotsuga, Tochigi, Japan

**Keywords:** *Staphylococcus aureus* bacteremia, Management, General internal medicine, Echocardiography

## Abstract

**Objectives:**

Consultation with infectious disease specialists is associated with reduced patient mortality in the care of patients with *Staphylococcus aureus* bacteremia (SAB) through appropriate management of complications including infective endocarditis. This study aimed to determine the rates of confirmation of a negative blood culture, implementation of echocardiography, and administration of appropriate antibiotics in patients with SAB at a university hospital in Japan that provides general internal medicine and not an infectious disease consultation service.

**Methods:**

We conducted a retrospective cohort study at Dokkyo Medical University Hospital in Japan. Patients eligible for inclusion in the study were ≥20 years of age with ≥1 positive blood culture for *S. aureus* identified in a clinical microbiology laboratory. The primary outcome was the proportion of patients with confirmation of a negative blood culture, implementation of echocardiography, and administration of appropriate antimicrobial agents.

**Results:**

A total of 109 patients with SAB were included in the analysis. Follow-up blood cultures were collected in 91 patients and negative results were documented in 88 patients. Follow-up blood culture collection was performed within 4 days of the initial blood culture collection in 49 patients. Echocardiography was performed appropriately in 40 patients. Appropriate antibiotic therapy was administered in 36 patients.

**Conclusions:**

Quality-of-care indicators were more commonly implemented in patients with SAB who received general internal medicine consultation than in those who did not.

## Introduction

*Staphylococcus aureus* is a common pathogen in community- and hospital-acquired infections, and is associated with considerable morbidity and mortality.^1^ *S. aureus* bacteremia (SAB) can lead to infective endocarditis and abscess formation in remote sites and is associated with a 30-day mortality rate as high as 20% even with appropriate antibiotic therapy.^2^ Despite medical advances, the mortality rate from SAB has not greatly improved since the 1990s.^2^ Consultation with infectious disease specialists in the care of patients with SAB is associated with reduced mortality of patients in multiple studies.^3–5^ The lower mortality rate of patients with SAB who receive infectious disease consultation is attributed to the following three factors: implementation of echocardiography, confirmation of negative blood cultures, and administration of appropriate antibiotics.^4–7^ These findings suggest that improving patients’ survival may be possible even if infectious disease consultation is not available, as long as these three factors are consistently implemented for patients with SAB. These three factors can be described as quality-of-care indicators.^4–7^ Although studies have compared the effect of algorithm-based and standard care on patients’ outcomes, infectious disease consultation is also performed in the majority of cases.^8^ Some studies have shown that these quality-of-care bundles have reduced mortality and recurrence rates in SAB.^6^

In Japan, because of the shortage of infectious disease specialists, infectious disease consultation is not available in most hospitals, including some university hospitals.^9^ Therefore, general internal medicine (GIM) physicians in Japan often provide consultation service for patients with complex infectious diseases such as SAB, although they do not have board certification in infectious diseases. In this context, medical consultation for conditions such as fever and antimicrobial selection is provided by physicians specializing in GIM at the hospital, guided by quality-of-care indicators, clinical guidelines, and textbooks.^10,11^

Currently, few studies have assessed quality-of-care indicators in the management of *S. aureus* bacteremia in Japanese hospitals, and studies in which infectious disease specialists did not intervene are scarce.^12^ In a study involving 10 Emergency Departments, no quality-of-care indicators were used in 86% of SAB cases.^12^

This study aimed to determine the rates of confirmation of a negative blood culture, implementation of echocardiography, and administration of appropriate antibiotics in patients with SAB at a university hospital in Japan that does not have an infectious disease consultation service, but has a GIM consultation service, and to assess patients’ outcomes.

## Methods

### Study design

We conducted a retrospective cohort study at Dokkyo Medical University Hospital (DMUH), which operates 1195 acute care beds, in Japan. DMUH does not have an infectious disease consultation service. Participants were enrolled between 1 April 2019 and 31 March 2021.

### Department of Diagnostic and Generalist Medicine at DMUH

The Department of Diagnostic and Generalist Medicine at DMUH covers GIM services, which include a medical outpatient clinic, urgent care clinic, and emergency medicine hospital medicine, and also provides medical consultation for patients with complex infection upon request.^10^ The Department of Diagnostic and Generalist Medicine at DMUH was founded in April 2016. At DMUH, GIM physicians do not routinely participate in the care of patients with SAB but provide a consultation service if consulted by the attending physicians of these patients.

### Patients

Hospitalized patients who were ≥20 years of age and had ≥1 positive blood culture for *S. aureus* collected during hospitalization or collected in an outpatient clinic, which led to the hospitalization, were eligible for the study. Patients who died within 2 days of the index blood culture collection were excluded because more than 90% of SAB results in a positive blood culture within 2 days.^13^ Additionally, patients who were transferred to other hospitals within 2 days and patients treated as outpatients were excluded.

### Definitions

The patients were classified as having hospital-acquired or non-hospital-acquired SAB^14^. Hospital-acquired SAB was defined as SAB for which index positive blood cultures were obtained from patients who had been hospitalized for more than 48 hours or from patients who had been hospitalized in another hospital and then transferred. Patients who did not meet the definition of hospital-acquired SAB were defined as having non-hospital-acquired SAB.^14^ Uncomplicated SAB was defined when all of the following criteria were met: 1) negative blood cultures were collected within 4 days of the index positive blood culture, 2) absence of findings suggestive of infective endocarditis at least once on transesophageal echocardiography (TEE) for non-hospital-acquired SAB or at least once on transthoracic echocardiography (TTE) for hospital-acquired SAB, 3) absence of distant lesions on a physical examination, and 4) absence of implanted intravascular devices.^1,2^ Complicated SAB was defined when any of these four criteria were not met. Appropriate antimicrobial therapy was defined when an appropriate agent was administered for a minimum of 2 weeks from the date of the first confirmed negative blood culture for uncomplicated SAB and a minimum of 4 weeks from the date of the first confirmed negative blood culture for complicated SAB. Patients who died in hospital were considered to have received appropriate antimicrobial therapy if they had received an appropriate agent up to the date of their death.

Appropriate antimicrobial agents included cefazolin, ceftriaxone, cefepime, and meropenem for methicillin-susceptible *S. aureus* (MSSA), and vancomycin, teicoplanin, and daptomycin for methicillin-resistant *S. aureus* (MRSA). Penicillin was not included among the appropriate agents because susceptibility to this agent was not routinely reported from the clinical microbiology laboratories.

Appropriate echocardiography was defined when TEE was performed in the presence of an implanted intravascular device or for any non-hospital-acquired SAB, or when TTE was performed for hospital-acquired SAB in the absence of an implanted intravascular device. GIM consultation was considered present if the patient was managed at least once by the Department of Diagnostic and Generalist Medicine after positive blood culture results became available during the course of the hospitalization.

The primary outcome was the proportion of patients with confirmation of a negative blood culture, implementation of echocardiography, and administration of appropriate antimicrobial agents based on the presence or absence of GIM consultation. The secondary outcome was in-hospital mortality based on the presence or absence of GIM consultation.

### Data collection

Data on the patients’ age, sex, and microbiology were initially extracted. Three investigators (SH, YH, and MY) then independently reviewed the patients’ electronic medical records and collected the following data: body mass index, onset location, date of blood culture collection, echocardiographic results, presence or absence of GIM consultation, survival in hospital, recurrence within 90 days, days of antimicrobial therapy, and the antimicrobials administered. Finally, the patients were classified into having complicated or uncomplicated SAB according to the above-mentioned definition, and any ambiguity was resolved by consensus among the investigators (SH, YH, and MY).

### Statistical analysis

Continuous variables are shown as the mean±standard deviation, and binary and categorical data are shown as the percentage. Continuous variables were compared using the Mann–Whitney U test, and binary and categorical variables were compared using Fisher’s test between groups with and without GIM consultation. Univariable logistic regression models were used to calculate the odds ratio of variables for outcomes. The statistical analysis was performed using EZR (version 1.61; Saitama Medical Center, Jichi Medical University, Saitama, Japan; based on R ver. 4.2.2).^15^

### Ethical considerations

Approval of the study was obtained from the institutional review board of DMUH (record number: R3-247). We followed the Declaration of Helsinki and the Strengthening the Reporting of Observational Studies in Epidemiology (STROBE) guidelines.^16^ Written informed consent was waived by the board under the condition that the opt-out method was used.

## Results

A total of 122 patients with SAB were enrolled during the study period. Of these, 11 patients died within 2 days of blood culture collection, one was transferred within 2 days, and one was treated as an outpatient. After excluding these 13 patients, 109 patients were included in the analysis. The mean age was 65.2 years (standard deviation 16.9 years), men accounted for 77/109 (65.1%) patients, and the mean body mass index was 22.1 kg/m^2^. In this cohort, 46 (42.2%) patients had non-hospital-acquired SAB and 63 (57.8%) had hospital-acquired SAB. MSSA was the culprit organism in 80 (73.4%) patients and MRSA in 29 (26.6%) patients. There were 96 (88.1%) patients with complicated SAB and 13 (11.9%) patients with uncomplicated SAB (Table 1).

Follow-up blood cultures were collected in 91 (83.5%) patients and negative results were documented in 88 (80.7%) patients. Follow-up blood culture collection was performed within 4 days of the initial blood culture collection in 49 (45%) patients, and these follow-up cultures were negative in 34 (31.2%) patients. TTE was performed at least once in 77 (70.6%) patients and TEE was performed at least once in 16 (14.7%) patients. TTE was not performed in 7 (15.2%) patients with non-hospital-acquired SAB and in 25 (39.7%) patients with hospital-acquired SAB. TEE was not performed in 33 (71.7%) patients with non-hospital-acquired SAB. Appropriate echocardiography was performed in 40 (36.7%) patients. Appropriate antimicrobial therapy was administered to 36 (33.0%) patients. There were 13 in-hospital deaths and 15 deaths within 90 days (Table 2).

When we compared patients for whom GIM was consulted (n=25) with those for whom GIM was not consulted (n=84), the baseline characteristics were not different between the two groups, except for the proportion of patients with non-hospital-acquired SAB. The proportion of patients with non-hospital-acquired SAB was higher in those seen by the GIM service than in those who did not use the GIM service (19/25, 76% vs. 27/84, 32%, P<.001) (Table 3). The proportions of patients with confirmation of a negative blood culture (25/25 patients, 100% vs. 63/84 patients, 75.0%, P=.006), implementation of TTE (23/25 patients, 92.0% vs. 54/84 patients, 64.3%, P=.006), and appropriate antimicrobial therapy (18/25 patients, 72.0% vs. 18/84 patients, 21.4%, P<.001) were significantly higher in patients who received GIM consultation than in those who did not receive GIM consultation. In-hospital death occurred in 3 (12.0%) patients with GIM consultation and in 10 (11.9%) patients without GIM consultation, with no significant difference between the two groups of patients. Recurrence within 90 days was also not different between the two groups (Table 4).

## Discussion

This study showed that at a university hospital without an infectious disease consultation service, the rates of confirmation of a negative blood culture, implementation of echocardiography, and administration of an appropriate antibiotic in patients with SAB were significantly higher in those who received GIM consultation than in those who did not.

The proportions of patients with confirmation of a negative blood culture and who underwent any echocardiography are comparable to those described in studies with infectious disease consultation.^4,5,13^ However, the proportion of patients who received appropriate treatment in this study is 40% lower than that in studies in which infectious disease consultation was available.^4,5,13^ Only 26% (12/46 patients) of patients with non-hospital-acquired SAB underwent TEE. This result is similar to that in a previous study, which mostly included patients with non-hospital-acquired SAB, and showed that TEE was performed in only 24% of SAB cases.^17^ Infective endocarditis is more common in non-hospital-acquired SAB than in hospital-acquired SAB.^18,19^ Additionally, TEE, which has a higher sensitivity of detecting structural abnormalities related to infective endocarditis than TTE, is recommended for patients with non-hospital-acquired SAB.^20–22^ Suboptimal implementation of TEE suggests that its indication may not have been adequately evaluated in these settings.^23,24^

Among the quality-of-care indicators for managing SAB, some studies have suggested that consultation with infectious disease specialists is the most effective in improving patients’ outcomes.^25^ However, there is a shortage of infectious disease specialists in Japan (0.9/100,000 people in Japan compared with 2.4/100,000 people in the USA).^26^ In fact, as of May 2023, there were only 1770 board-certified infectious disease specialists in Japan (1.4/100,000 people).^9^ Bundled approaches to SAB,^6,27^ although less effective than infectious disease consultation, have also been attempted and may improve outcomes for patients with SAB. In our study, the rates of confirmation of a negative blood culture, implementation of TTE, and appropriate antimicrobial administration were higher in patients who were seen by the GIM service than in those who were not. Our study and a previous study showed that patients with GIM consultation were almost as likely to have blood cultures drawn for confirmation of culture negativity as those with infectious diseases consultation.^5^ Additionally, in our study and this previous study, the rate of echocardiographic implementation was higher in the GIM consultation group but the percentage of appropriate antimicrobial therapy was lower in the GIM consultation group than in the infectious disease specialist consultation group.^5^ The reason why GIM physicians are able to perform well in implementing essential interventions may be because GIM physicians, who generally serve as hospitalists in Japan, tend to be familiar with practice guidelines and are likely to follow them in clinical care.^28–30^ However, there appear to be opportunities for GIM physicians to further improve the appropriateness of antimicrobial therapy compared with infectious disease specialists. Indeed, follow-up blood cultures within 4 days and implementation of TTE, which are recommended by the MRSA infection guidelines by the IDSA,^1^ were also significantly more common in patients with GIM consultation than in those without GIM consultation in this study. This finding suggests that GIM consultation may be a way of providing appropriate treatment for SAB in hospitals where infectious disease specialists are not available.

This study has several limitations. First, because this study focused on the treatment of SAB, the duration of treatment was defined only based on complicated and uncomplicated SAB and did not evaluate other conditions that may require longer treatment, such as prosthetic valve endocarditis. Second, the duration of antimicrobial therapy may have been underestimated because the duration of appropriate therapy did not include patients who were switched to agents that are not considered standard for SAB but still have coverage against organisms (e.g., ampicillin-sulbactam for patients who had pneumonia in addition to SAB by MSSA). Third, TTE by itself cannot be used to assess the quality of a patient’s care. However, the implementation of TTE in patients with SAB suggests that infectious endocarditis was considered and assessed. Fourth, because this was a single-center study, the findings may not be generalizable to comparable patients at other hospitals. In the present study, the mortality and recurrence rates were similar, regardless of the presence or absence of GIM consultation. This finding may have been due to unmeasured differences in patients’ care between the specialties of infectious diseases and GIM or the relatively small number of patients included in this study.

## Conclusion

Quality-of-care indicators in the care of patients with SAB were more commonly implemented in patients who received GIM consultation than in those who did not. This finding suggests that quality of care may be improved in patients with SAB and GIM consultation even when infectious disease consultation is not available.

## Figures and Tables

**Table1:** Baseline characteristics of the patients with SAB

	All patients (n=109)
Age, years	65.2±16.9
Male sex	71 (65.1)
BMI, kg/m^2^	22.1±4.9
Non-hospital-acquired SAB	46 (42.2)
MSSA	80 (73.4)
Complicated SAB	96 (88.1)

Data are the mean±standard deviation or n (%). SAB, *Staphylococcus aureus* bacteremia; BMI, body mass index; MSSA, methicillin-susceptible *Staphylococcus aureus*.

**Table2:** Outcomes of the patients with SAB

	All patients (n=109) unless otherwise specified
Negative blood culture documented	88 (80.7)
Follow-up blood culture collected within 4 days	49 (45.0)
TTE implemented	77 (70.6)
Echocardiography performed appropriately	40 (36.7)
TEE for patients with non-hospital-acquired SAB	12/46 (26.1)
Appropriate antimicrobial therapy provided	36 (33.0)
Death in hospital	13 (11.9)
Recurrence 90 days after completion of antimicrobial therapy	6/98 (6.1)
Death within 90 days of the first positive blood culture	15/99 (15.2)

Data are n (%). SAB, *Staphylococcus aureus* bacteremia; TTE, transthoracic echocardiography; TEE, transesophageal echocardiography.

**Table3:** Baseline characteristics of patients with SAB with or without GIM consultation

	With GIM consultation (n=25)	Without GIM consultation (n=84)	P value
Age, years	69.3±14.6	64.0±17.4	.19
Male sex	14 (56.0)	57 (67.9)	.34
BMI, kg/m^2^	22.0±4.5	22.2±5.0	.99
Non-hospital-acquired SAB	19 (76.0)	27 (32.1)	<.001
MSSA	17 (68.0)	63 (75.0)	.61
Complicated SAB	22 (88.0)	74 (88.1)	>.99

Data are the mean±standard deviation or n (%). SAB, *Staphylococcus aureus* bacteremia; GIM, general internal medicine; BMI, body mass index; MSSA, methicillin-susceptible *Staphylococcus aureus*.

**Table4:** Outcomes of patients with SAB with or without GIM consultation

	With GIM consultation (n=25)	Without GIM consultation (n=84)	Odds ratio (95% CI)	P value
Negative blood culture documented	25 (100)	63 (75.0)	1.33 (1.18–1.51)	.006
Follow-up blood culture collected within 4 days	21 (84.0)	28 (33.3)	2.52 (1.78–3.57)	<.001
TTE implemented	23 (92.0)	54 (64.3)	1.43 (1.18–1.74)	.006
Echocardiography implemented appropriately	11 (44.0)	29 (34.5)	1.27 (0.75–2.17)	.48
TEE for patients with non-hospital-acquired SAB	9/20 (45)	3/26 (11.5)	3.90 (1.21–12.56)	.010
Appropriate antimicrobial therapy provided	18 (72.0)	18 (21.4)	3.36 (2.09–5.41)	<.001
Death in hospital	3 (12.0)	10 (11.9)	1.01 (0.30–3.38)	>.99
Recurrence 90 days after completion of antimicrobial therapy	1/23 (4.3)	5/75 (6.7)	0.65 (0.08–5.30)	.69
Death within 90 days of the first positive blood culture	3/22 (13.6)	12/77 (15.6)	0.88 (0.27–28.3)	>.99

Data are n (%). SAB, *Staphylococcus aureus* bacteremia; GIM, general internal medicine; TTE, transthoracic echocardiography; TEE, transesophageal echocardiography.

## References

[1] Liu C, Bayer A, Cosgrove SE, Daum RS, Fridkin SK, Gorwitz RJ, Kaplan SL, Karchmer AW, Levine DP, Murray BE, Rybak MJ, Talan DA, Chambers HF. Clinical Practice Guidelines by the Infectious Diseases Society of America for the Treatment of Methicillin-Resistant Staphylococcus aureus Infections in Adults and Children. Clin Infect Dis 2011; 52: e18–e55. 21208910 10.1093/cid/ciq146

[2] Holland TL, Arnold C, Fowler VG Jr. Clinical Management of Staphylococcus aureus Bacteremia. JAMA 2014; 312: 1330–1341. 25268440 10.1001/jama.2014.9743 PMC4263314

[3] Honda H, Krauss MJ, Jones JC, Olsen MA, Warren DK. The value of infectious diseases consultation in Staphylococcus aureus bacteremia. Am J Med 2010; 123: 631–637. 20493464 10.1016/j.amjmed.2010.01.015 PMC3606273

[4] Bai AD, Showler A, Burry L, Steinberg M, Ricciuto DR, Fernandes T, Chiu A, Raybardhan S, Science M, Fernando E, Tomlinson G, Bell CM, Morris AM. Impact of Infectious Disease Consultation on Quality of Care, Mortality, and Length of Stay in Staphylococcus aureus Bacteremia: Results from a Large Multicenter Cohort Study. Clin Infect Dis 2015; 60: 1451–1461. 25701854 10.1093/cid/civ120

[5] Vogel M, Schmitz RPH, Hagel S, Pletz MW, Gagelmann N, Scherag A, Schlattmann P, Brunkhorst FM. Infectious disease consultation for staphylococcus aureus bacteremia—a systematic review and meta-analysis. J Infect 2016; 72: 19–28. 26453841 10.1016/j.jinf.2015.09.037

[6] López-Cortés LE, Del Toro MD, Gálvez-Acebal J, et al. Impact of an evidence-based bundle intervention in the quality-of-care management and outcome of staphylococcus aureus bacteremia. Clin Infect Dis 2013; 57: 1225–1233. 23929889 10.1093/cid/cit499

[7] Lam JC, Stokes W. The Golden Grapes of Wrath—Staphylococcus aureus Bacteremia: A Clinical Review. Am J Med 2023; 136: 19–26. 36179908 10.1016/j.amjmed.2022.09.017

[8] Holland TL, Raad I, Boucher HW, et al. Effect of Algorithm-Based Therapy Vs Usual Care on Clinical Success and Serious Adverse Events in Patients with Staphylococcal Bacteremia. JAMA 2018; 320: 1249–1258. 30264119 10.1001/jama.2018.13155 PMC6233609

[9] Semmoni Meibo (Infectious Disease Specialist Directory); 2024 (in Japanese). <https://www.kansensho.or.jp/modules/senmoni/index.php? content_id=29> (Apr 15, 2024).

[10] Yokose M, Harada Y, Hanai S, Tomiyama S, Shimizu T. Outcomes of General Internal Medicine Consultations for Diagnosis from Specialists in a Tertiary Hospital: A Retrospective Observational Study. Int J Gen Med 2022; 15: 7209–7217. 36124102 10.2147/IJGM.S378146 PMC9482410

[11] Kawamura R, Harada Y, Yokose M, Hanai S, Suzuki Y, Shimizu T. Survey of Inpatient Consultations with General Internal Medicine Physicians in a Tertiary Hospital: A Retrospective Observational Study. Int J Gen Med 2023; 16: 1295–1302. 37081930 10.2147/IJGM.S408768 PMC10112478

[12] Miyamoto K, Kato S, Kitayama J, Okawa J, Okamoto A, Kamei J, Yoshiya K, Asai H, Adachi S, Yukioka H, Akimoto H, Okuchi K. Adherence rate of quality-of-care indicators for Staphylococcus aureus bacteremia is extremely low in Japanese emergency and critical care departments: a multicenter retrospective observational study. Acute Med Surg 2017; 5: 140–145. 29657725 10.1002/ams2.316 PMC5891101

[13] Jenkins TC, Price CS, Sabel AL, Mehler PS, Burman WJ. Impact of Routine Infectious Diseases Service Consultation on the Evaluation, Management, and Outcomes of Staphylococcus aureus Bacteremia. Clin Infect Dis 2008; 46: 1000–1008. 18444816 10.1086/529190

[14] Friedman ND, Kaye KS, Stout JE, McGarry SA, Trivette SL, Briggs JP, Lamm W, Clark C, MacFarquhar J, Walton AL, Reller LB, Sexton DJ. Health Care-Associated Bloodstream Infections in Adults: A Reason to Change the Accepted Definition of Community-Acquired Infections. Ann Intern Med 2002; 137: 791–797. 12435215 10.7326/0003-4819-137-10-200211190-00007

[15] Kanda Y. Investigation of the Freely Available Easy-to-Use Software ‘Ezr’ for Medical Statistics. Bone Marrow Transplant 2013; 48: 452–458. 23208313 10.1038/bmt.2012.244 PMC3590441

[16] von Elm E, Altman DG, Egger M, Pocock SJ, Gøtzsche PC, Vandenbroucke JP. The Strengthening the Reporting of Observational Studies in Epidemiology (Strobe) Statement: Guidelines for Reporting Observational Studies. Ann Intern Med 2007; 147: 573–577. 17938396 10.7326/0003-4819-147-8-200710160-00010

[17] Young H, Knepper BC, Price CS, Heard S, Jenkins TC. Clinical Reasoning of Infectious Diseases Physicians Behind the Use or Nonuse of Transesophageal Echocardiography in Staphylococcus aureus Bacteremia. Open Forum Infect Dis 2016; 3: ofw204. 27833929 10.1093/ofid/ofw204 PMC5102142

[18] Rasmussen RV, Høst U, Arpi M, et al. Prevalence of infective endocarditis in patients with Staphylococcus aureus bacteraemia: the value of screening with echocardiography. Eur J Echocardiogr 2011; 12: 414–420. 21685200 10.1093/ejechocard/jer023 PMC3117467

[19] Le Moing V, Alla F, Doco-Lecompte T, Delahaye F, Piroth L, Chirouze C, Tattevin P, Lavigne J-P, Erpelding M-L, Hoen B, Vandenesch F, Duval X. Staphylococcus aureus Bloodstream Infection and Endocarditis - a Prospective Cohort Study. PLoS One 2015; 10: e0127385. 26020939 10.1371/journal.pone.0127385 PMC4447452

[20] Palraj BR, Baddour LM, Hess EP, Steckelberg JM, Wilson WR, Lahr BD, Sohail MR. Predicting Risk of Endocarditis Using a Clinical Tool (Predict): Scoring System to Guide Use of Echocardiography in the Management of Staphylococcus aureus Bacteremia. Clin Infect Dis 2015; 61: 18–28. 25810284 10.1093/cid/civ235 PMC4542912

[21] Abu Saleh O, Fida M, Asbury K, Narichania A, Sotello D, Bosch W, Vikram HR, Palraj R, Lahr B, Baddour LM, Sohail MR. Prospective Validation of Predict and Its Impact on the Transesophageal Echocardiography Use in Management of Staphylococcus aureus Bacteremia. Clin Infect Dis 2021; 73: e1745–e1753. 32569366 10.1093/cid/ciaa844 PMC8492221

[22] Tubiana S, Duval X, Alla F, Selton-Suty C, Tattevin P, Delahaye F, Piroth L, Chirouze C, Lavigne JP, Erpelding ML, Hoen B, Vandenesch F, Iung B, Le Moing V. The VIRSTA score, a prediction score to estimate risk of infective endocarditis and determine priority for echocardiography in patients with Staphylococcus aureus bacteremia. J Infect 2016; 72: 544–553. 26916042 10.1016/j.jinf.2016.02.003

[23] Sekar P, Johnson JR, Thurn JR, Drekonja DM, Morrison VA, Chandrashekhar Y, Adabag S, Kuskowski MA, Filice GA. Comparative Sensitivity of Transthoracic and Transesophageal Echocardiography in Diagnosis of Infective Endocarditis among Veterans with Staphylococcus aureus Bacteremia. Open Forum Infect Dis 2017; 4: ofx035. 28470017 10.1093/ofid/ofx035 PMC5407216

[24] Heriot GS, Tong SYC, Cheng AC, Liew D. A Scenario-Based Survey of Expert Echocardiography Recommendations for Patients with Staphylococcus aureus Bacteremia at Varying Risk for Endocarditis. JAMA Netw Open 2020; 3: e202401. 32271391 10.1001/jamanetworkopen.2020.2401 PMC7146099

[25] Lam JC, Gregson DB, Robinson S, Somayaji R, Welikovitch L, Conly JM, Parkins MD. Infectious diseases consultation improves key performance metrics in the management of Staphylococcus aureus bacteremia: A multicentre cohort study. J Assoc Med Microbiol Infect Dis Can 2019; 4: 24–32. 36338780 10.3138/jammi.2018-0036 PMC9603189

[26] Iwata K. Quantitative and Qualitative Problems of Infectious Diseases Fellowship in Japan. Int J Infect Dis 2013; 17: e1098–e1099. 24075398 10.1016/j.ijid.2013.07.009

[27] Green J, Howard J, Shankar A, Clinghan R, Luff T, Birch M, Pithie A, Werno A, Metcalf S, Chambers S. Assessing the impact of a ‘bundle of care’ approach to Staphylococcus aureus bacteraemia in a tertiary hospital. Infect Prev Pract 2020; 2: 100096. 34368726 10.1016/j.infpip.2020.100096 PMC8336039

[28] McCulloh RJ, Smitherman S, Adelsky S, Congdon M, Librizzi J, Koehn K, Alverson B. Hospitalist and Nonhospitalist Adherence to Evidence-Based Quality Metrics for Bronchiolitis. Hosp Pediatr 2012; 2: 19–25. 24319809 10.1542/hpeds.2011-0002-2

[29] Hamada O, Tsutsumi T, Imanaka Y. Efficiency of the Japanese Hospitalist System for Patients with Urinary Tract Infection: A Propensity-Matched Analysis. Intern Med 2023; 62: 1131–1138. 36070954 10.2169/internalmedicine.8944-21 PMC10183293

[30] Tago M, Watari T, Shikino K, Sasaki Y, Takahashi H, Shimizu T. Five Tips for Becoming an Ideal General Hospitalist. Int J Gen Med 2021; 14: 10417–10421. 35002297 10.2147/IJGM.S341050 PMC8722286

